# P-878. Early intravenous-to-oral antibiotic conversions for community-acquired pneumonia across an integrated healthcare system

**DOI:** 10.1093/ofid/ofaf695.1086

**Published:** 2026-01-11

**Authors:** Daniel J Livorsi, Logan Daniels, Bruce Alexander, Brett Heintz, Brian Lund

**Affiliations:** University of Iowa Carver College of Medicine, Iowa City, IA; Iowa City VA Health Care System, Iowa City, Iowa; Iowa City VA Medical Center, Iowa City, Iowa; Iowa City VA Medical Center, Iowa City, Iowa; Iowa City VA Health Care System, Iowa City, Iowa

## Abstract

**Background:**

Early conversions from intravenous (IV) to oral antibiotics have been proposed as a target for antibiotic stewardship programs, but it is unclear how often these conversions are actually performed

**Figure:**
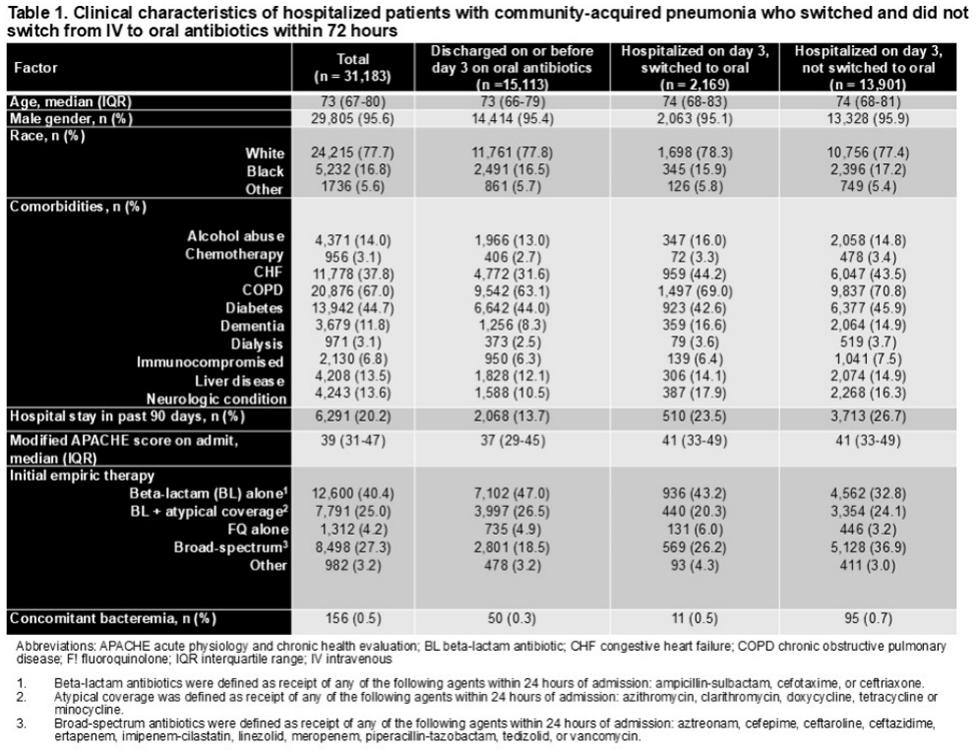

**Figure:**
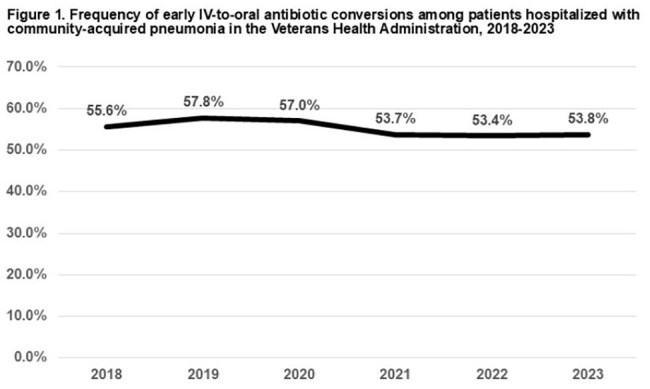

**Methods:**

We performed a retrospective cohort study to measure the frequency that patients with community-acquired pneumonia (CAP) had an early IV to oral antibiotic conversion, i.e. within 72 hours of admission. All acute-care admissions during 2018-2023 to Veterans Health Administration (VHA) hospitals were included. Patients were ineligible for switching if they were in the intensive care unit, not taking other oral medications, or were diagnosed with another infection. The secondary outcome was death and/or hospital readmission within 30 days of discharge. To account for the possibility that hospitals with lower switch rates had a different patient case-mix, we calculated an expected switch rate at each facility by using patient-level variables in a log-binomial model. Hospitals were grouped into quartiles based on their observed-to-expected (O:E) ratios and the secondary outcome was compared across quartiles using a Kruskal Wallis test.

**Figure:**
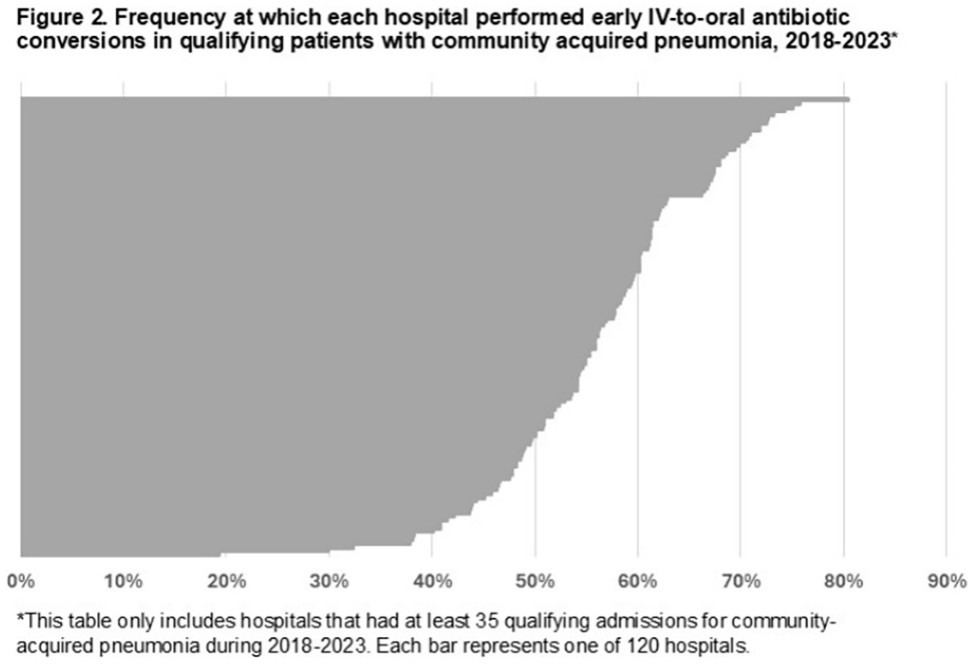

**Figure:**
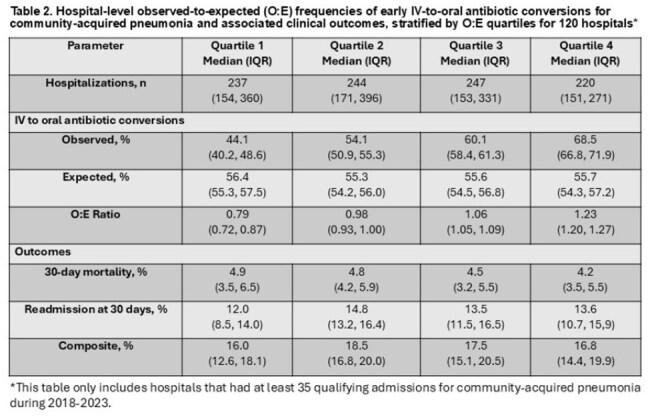

**Results:**

There were 31,183 admissions that met criteria across 124 VHA hospitals (Table 1). Median age was 73 years (IQR 67-80); 29,805 (96%) were male. There were 17,282 (55%) patients who were switched to oral antibiotics by day 3. Among 16,070 patients still in the hospital on day 3, only 2169 (13.5%) were switched. The hospital-level median switch rate was 57% (IQR 50-62%), and the frequency of switching did not change over time (Figures 1 and 2). The O:E ratio for switches ranged from 0.79 among hospitals in the lowest quartile to 1.23 in the highest quartile. Overall, 5,629 (18.1%) patients died and/or were re-admitted within 30 days. There was no difference in this composite outcome across quartiles (Kruskal-Wallis χ^2^ =5.4; p=0.14) (Table 2).

**Conclusion:**

Early conversions from IV-to-oral antibiotics for patients hospitalized with CAP occurred in approximately half of eligible cases. Outcomes among patients at hospitals with high conversion rates were comparable to outcomes at hospitals with low rates, thereby supporting the safety of early conversions. More concerted efforts to promote these conversions, when appropriate, are needed.

**Disclosures:**

All Authors: No reported disclosures

